# Coffee brewing sonoreactor for reducing the time of cold brew from several hours to minutes while maintaining sensory attributes

**DOI:** 10.1016/j.ultsonch.2024.106885

**Published:** 2024-04-23

**Authors:** Shih-Hao Chiu, Nikunj Naliyadhara, Martin P. Bucknall, Donald S. Thomas, Heather E. Smyth, Jaqueline M. Nadolny, Kourosh Kalantar-Zadeh, Francisco J. Trujillo

**Affiliations:** a School of Chemical Engineering, University of New South Wales, Sydney 2052, NSW, Australia; b Bioanalytical Mass Spectrometry Facility, Mark Wainwright Analytical Centre, University of New South Wales, Sydney 2052, NSW, Australia; c NMR Facility, Mark Wainwright Analytical Centre, University of New South Wales, Sydney 2052, NSW, Australia; d Centre for Nutrition and Food Sciences, Queensland Alliance for Agriculture and Food Innovation, The University of Queensland, Brisbane 4072, Queensland, Australia; e School of Chemical and Biomolecular Engineering, The University of Sydney, Sydney 2008, NSW, Australia

**Keywords:** Sonoreactor, Coffee brewing, Ultrasonication, Cavitation modelling, Food processing

## Abstract

This research designed and developed an ultrasonic reactor for a fast and on demand production of cold brew coffee, remarkably reducing the brewing time from 24 h to less than 3 min. The technology was engineered by utilizing resonance to induce ultrasonic waves around the walls of the brewing basket of an espresso machine. The sound transmission system comprised a transducer, a horn and a brewing basket. This arrangement transformed the coffee basket into an effective sonoreactor that injected sound waves at multiple points through its walls, thereby generating multiple regions for acoustic cavitation within the reactor. Furthermore, acoustic streaming induced greater mixing and enhanced mass transfer during brewing. The design was accomplished by modeling the transmission of sound, and acoustic cavitation. Brew characterization and chemical composition analysis was performed, considering factors such as pH, acidity, color, and the composition of caffeine, fatty acids, and volatiles. The efficiency of the extraction increased by decreasing the basket loading percentage (BLP). For instance, sonicating at 100 W doubled the extraction yield and caffeine concentration, from 15.05 % to 33.44 % at BLP = 33 %, and from 0.91 mg/mL to 1.84 mg/mL at BLP = 67 %, respectively. The total fatty acids increased from 1.16 mg/mL to 9.20 mg/mL, representing an eightfold increase, at BLP = 33 %. Finally, a sensory analysis was conducted to evaluate appearance, aroma, texture, flavor, and aftertaste, which demonstrated that coffee brewed for 1 and 3 min in the sonoreactor exhibited almost undistinguishable properties compared to a standard 24 h brewing without ultrasound.

## Highlights

• The sonoreactor reduced the cold brew time from 24 h to less than 3 min.
• Vibrations through the wall of the sonoreactor generated multiple cavitation zones.
• Acoustic cavitation significantly increased the extraction yield.
• Decreasing the basket loading percentage (BLP) increased extraction yield.
• It produced coffee with sensory qualities similar to those of 24-hour cold brew.

## 1 Introduction

Coffee is one of the most consumed and cherished beverages worldwide, playing a central role in the diets and social interactions of most consumers, with its aroma being universally appreciated [1]. Brewing coffee involves a meticulous process including roasting, grinding, extracting with water, and filtering [2]. Coffee is comprised of about 2,000 chemical compounds including carbohydrates, lipids, vitamins, minerals, phenolic compounds, pyrazines, and furans, among others [3], some of which are volatile, and others are non-volatile. The volatile compounds are responsible for the distinctive aroma of coffee, while the non-volatiles influence its taste, body, bitterness, and acidity. Key non-volatile components include caffeine, renowned for its stimulating effects, and oils, both impacting the sensory experience and functional properties of coffee. Coffee beans contain between 7 % to 17 % lipids, primarily consisting of triacylglycerols, sterols, and tocopherols [4]. This mixture of compounds is responsible for the sensory complexity of coffee brews.

Coffee enthusiasts continually seek premium coffee beverages, driven by a desire for brews with distinct sensory qualities. Cold brew coffee, with its reduced acidity, enhanced sweetness, and floral aroma [5], [6], has recently surged in popularity. It is estimated that the cold brew market will continue to grow, capturing a significant portion of the coffee market. Cold brew coffee is mostly produced by steeping coffee grounds in water at temperatures equal to or lower than ambient for a period ranging from 12 to 24 h [7]. Most of the market currently produces cold brew by using two vessels with a capacity of 1 to 5 L in size, with long brew times from 18 to 24 h. With the projected growing demand within the next 5 years, cafes and restaurants will require full-time brewers with capacities of 20–30 L each, and larger refrigeration space, which is prohibitive for most cafes. Hence, more continuous/rapid extraction methods will be needed to keep up with the strong demand.

Ultrasounds accelerate extraction processes due to acoustic cavitation [8], [9]. When acoustic bubbles, also called inertial bubbles, collapse near solid materials, such as coffee grounds, they generate micro-jets with the force to fracture the cell walls of plant tissues, intensifying the extraction of the intracellular content [10]. Recent studies have explored ultrasound's potential to enhance and expedite coffee brewing, particularly in cold brew preparations. For instance, Ahmed *et al.* [11] investigated the combined effect of ultrasonication and mixing, observing a significant increase in total soluble solids, phenol, and flavonoid content. Zamanipoor *et al.* [12] demonstrated that ultrasound enhanced coffee oil extraction, resulting in brews with sharper caramel tones, and elevated levels of key volatile compounds, thereby intensifying aroma and flavour profiles. Zhai *et al.* [13] explored the feasibility of accelerating coffee cold brew production using ultrasound, achieving a reduction of brewing time to just 1 h while simultaneously increasing total dissolved solids, total lipids, proteins, and tritrated acids. Importantly, ultrasound preserved cold brew flavour characteristics, including caramel, nutty, roasty and sweet aromas. Further research by Duagjai *et al.* [14] concluded that ultrasound facilitated the extraction of caffeine and polyphenols, offering a method to tailor brew characteristics without compromising antioxidant activity. Putro *et al.* [15] reported optimal conditions for ultrasound assisted coffee brewing, achieving a high total phenolic, flavonoid, and caffeine content at a temperature of 25 °C and brewing time of 15 min. It is worth noting that while these studies utilized traditional ultrasonic bath and horns, which are generally not practical for commercial implementations, and with long sonication durations being a hurdle for eventual consumer uptake, they revealed the potential of ultrasound technology as transformative for coffee brewing processes.

Despite extensive research into ultrasound’s potential for various food processing applications [12], progress towards market implementation has been sluggish due to challenges such as inadequate equipment design tailored for real-world food processing needs. This includes ensuring compliance with food safety regulations and achieving process scalability. These hurdles are compounded by a limited understanding of complex cavitation events and the lack of accurate computational models [16]. Fortunately, the capability of mathematical models to predict the interactions of cavitation bubbles and acoustic fields has recently advanced, evolving from simple linear models [17], which traditionally underpredict the sound attenuation on cavitating systems by orders of magnitude [18], to simple but powerful nonlinear Helmholtz equations. These equations are capable of more closely predicting the interaction of inertial bubbles with acoustic fields, although not perfectly [19], [20], [21].

To address the challenges that the increasing demand for cold brew will pose to cafes and restaurants in the near future, we designed and developed an ultrasonic coffee brewing system, consisting of a patented sound transmission system [22] that connects a bolt clamped Langevin transducer with a coffee brewing basket of a standard espresso machine via a metallic horn. This transformed the coffee basket into a powerful ultrasonic vessel, characterized by acoustic streaming, resulting in a very fast and efficient brewing with an outstanding reduction of the brewing time, from several hours to 3 min or less. Despite this drastic reduction, the resulting brews maintained nearly undistinguishable sensory attributes compared to a standard 24-hour cold brew, all while ensuring compliance with food safety standards, scalability, and ease of operation.

The design was accomplished by modelling the coupling of the acoustic vibration, piezoelectric effect, acoustic cavitation, and acoustic streaming. The physical properties of coffee brews, including pH, total titratable acidity, color, extraction yields, as well as the content of caffeine, fatty acids, and volatiles, were quantified, comparing sonicated and unsonicated samples. Finally, a sensory analysis with sensory panellists, trained in coffee, was conducted to compare the ultrasonic brews with a traditional 24-hour cold brew.

## 2 Methods

### 2.1 Design of ultrasonic coffee brewing machine

**Fig. 1:**
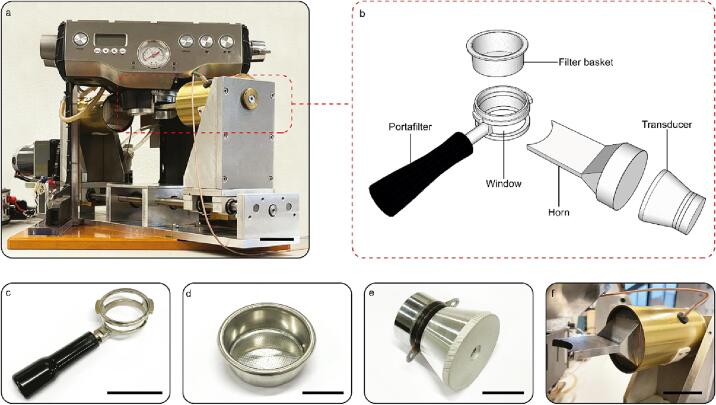
Customized ultrasonic coffee brewing machine. a) Photograph of the machine. b) Schematic of the parts for the ultrasound transmission into the coffee basket. c-f), Photographs of the portafilter (with corresponding window), filter basket, transducer, and the horn, respectively. (scale bar: 4 cm).

A Breville Dual Boiler BES920 expresso machine (shown in Fig. 1a,b) was modified to sonicate during brewing by placing a transducer-horn assembly comprising a 28 kHz bolt clamped Langevin transducer (Hesentec HS-4SH-4528 transducer) (Fig. 1e) and a horn (Fig. 1f). The horn transmits ultrasound from the transducer to the filter basket. The espresso machine comprised a housing having a group head, a showerhead, and a portafilter with a portafilter head (Fig. 1c) for carrying a coffee filter basket (Fig. 1d). A Fluid-o-Tech GA rotary vane pump with a 24 V DC brushless motor was used to pump water into the coffee basket. The original boiler of the Breville machine was used, but it was independently controlled with an integrated circuit. This control allowed managing the temperature of the water in the boiler, the temperature of the shower head, the revolutions per minute (RPM), and the time for the operation of the pump. A user interface programmed in Python was developed and connected to a laptop to control the brewing parameters.

The portafilter can engage and disengage with the group head, as normally done during the brewing of espressos. The portafilter head has a window in the side wall, permitting the horn to pass through the portafilter to contact the filter basket. Ultrasound was generated by the transducer, transmitted through the horn, and injected into the coffee basket through its walls. The transducer-horn assembly is movably mounted to the housing, allowing it to move between a storage position—enabling the portafilter head to be engaged or disengage with the group head— and a brewing position. In the brewing position, the transducer-horn assembly passes through the windows of the portafilter to contact the filter basket, performing sonication when the portafilter is engaged with the group head. The horn has a concave outer surface (Fig. 1f) that is complementary in shape to a section of the wall of the filter basket. The horn is moved in and out of engagement with the filter basket with a motor. The horn was designed to resonate with the transducer and the coffee basket, and it can be operated as a wavelength or a multiple of a half wavelength resonator.

### 2.2 Coffee shot preparation

The coffee shots were prepared from Campos Coffee's Caramelly & Rich Blend, which consists of medium roast arabica coffee beans. This blend combines fresh, high-quality specialty coffee beans from Ethiopia, Kenya, and Colombia, and the roasted beans deliver sweet caramel, butterscotch, and milk chocolate flavours. The beans were freshly ground with a Baratza Sette 270Wi grinder. The grinder can adjust the coffee's coarseness and fineness manually with a setting from 1 to 31, where 1 means very fine and 31 is very coarse. For ultrasonic brews, we used the setting of 3, which makes ground coffee as fine as in espressos, while 23 was used for making traditional cold brew coffee. Coffee grounds were weighted in the range of 6 g to 18 g in a filter basket before being placed in the portafilter, which was then connected to the group head of the coffee machine. Two different filter baskets were used, an 18 g 2-cup single-wall Breville basket (58 mm) and a 25 g ridgeless VST coffee basket (58 mm). Once the portafilter was positioned in the group head, the transducer-horn assembly was moved with a motor through the window in the portafilter head and into engagement with the filter basket. In the engaged position, the transducer-horn assembly applies gentle pressure to the filter basket.

**Fig. 2:**
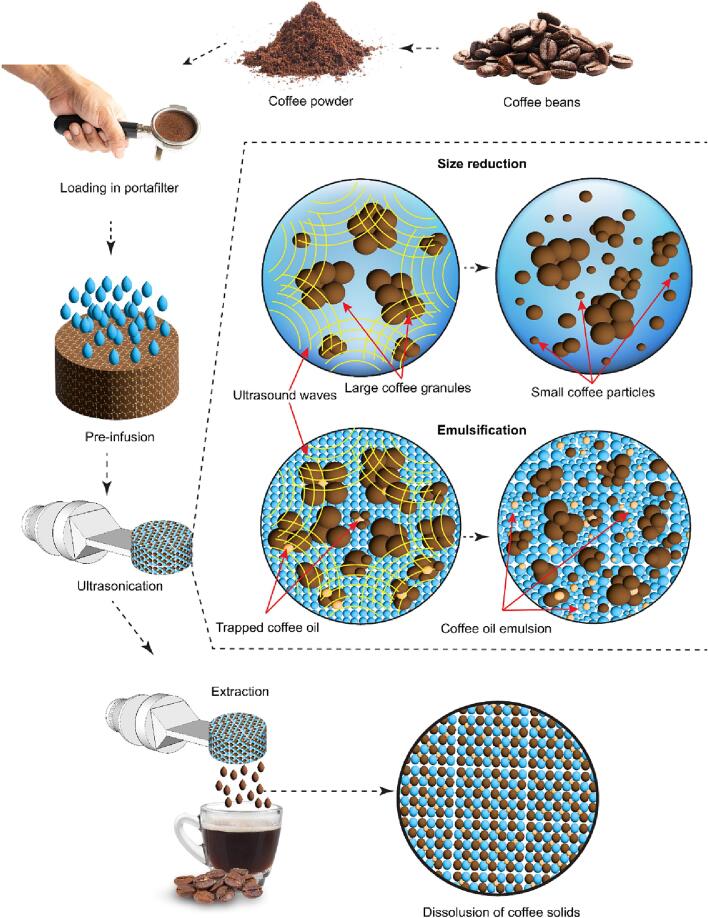
Illustration of the multifaceted effects of ultrasound on the coffee brewing process, encompassing particle size reduction, coffee oil emulsification, dissolution, and homogenization of fluid constituents. Illustration was generated with Adobe software (©Siravich/stock.adobe.com; ©saneh/stock.adobe.com; ©Rung/stock.adobe.com; ©phive2015/stock.adobe.com).

Once the transducer-horn assembly was engaged, water was pre-infused into the filter basket for about 4 s with a pump speed of 700 rpm. The water temperature can be set from ambient to 95 °C but the majority of the experiments used room temperature water to produce cold brew. When properly connected, the portafilter head sealed the contents of the filter basket, with back pressure retaining most of the liquid in the filter basket during brewing. Sonication was then applied with a power of 100 W via the engaged transducer-horn assembly to produce the brewed coffee. Our built transducer-horn assembly can be operated at frequencies of 27.2 kHz and 38.8 kHz, but the latter was used due to its quieter performance. Depending on the desired properties of the brew, water may be pumped to release the brew following various patterns, such as extracting the brewed coffee at the end of the sonication cycle or with various pump injections at different times during sonication. Cycles of sonication followed by water pumping can also be programmed for longer shots with higher brew ratios. Brew samples were collected in clean glass vials. Once the brewed coffee extraction was completed, the transducer-horn assembly was moved from the brewing position to the storage position, so, the portafilter containing the spent coffee grounds was removed. Control samples were prepared similarly to sonicated samples but without applying ultrasound. The main steps of the coffee brewing process are depicted on the top and left-hand side of Fig. 2.

### 2.3 Simulation and modelling

In 2022, Igor Garcia-Vargas *et al.* developed a comprehensive sonoreactor computational model in response to the limited predictive tools hindering the progress of ultrasound industrial applications [16]. In this study, we adopted a similar approach to model the nonlinear sound transmission in our system, the vibration through the horn and filter basket, and the piezoelectric effect on the transducer. In addition, we simulated the acoustic streaming force generated in the coffee basket, crucial for accelerating brewing through effective mixing. The model was implemented in COMSOL Multiphysics version 6.2.

#### 2.3.1 Vibration and piezoelectric effect modeling

The motion of linear elastic materials in time-harmonic independent form is expressed as

(1) T=cS

where T the stress tensor, S the strain tensor and c the elasticity tensor. The equation was solved for the horn (made of aluminium), the filter basket (constructed from stainless steel), and the metallic parts of the transducer (comprising an aluminium front driver and a stainless-steel back driver). Eq. (1) is extended to piezoelectric materials by adding electrostatics terms:

(2) $$T=cS+e^{t}E$$

(3) $$D=eE+{∊}^{s}E$$

The piezoelectric constants c, $e^{t}$ e and ${∊}^{s}$ are material properties expressed as tensors relating the stress and strain to the dielectric displacement D and the electric field E=∇V. In the piezoceramic (PZT-4), the Gauss law was solved $=\nabla D=\rho_{v}$, where $\rho_{v}$ is the volumetric density of charge. The piezoelectric sandwich of the transducer was modelled by setting the voltage in the active electrode and zero voltage in the grounded electrodes. More details are given by Garcia-Vargas *et al.* [16].

#### 2.3.2 Nonlinear Helmholtz equation for sound propagation and bubble dynamics model

Time-independent models of sound waves moving through bubbly liquids (mixtures of liquids and bubbles) are based on the Helmholtz equation formulated by Commander and Prosperetti [17]. This is a model that assumes small and linear oscillations of bubbles, induced by the sound field. The model is correct for sound waves with low-pressure amplitudes, interacting with small bubbles at low concentrations in liquids, resulting in small linear oscillations of the bubble around the equilibrium radius. However, it underpredicts the sound attenuation in cavitating systems by orders of magnitude [18] due to the high acoustic pressure amplitude required for cavitation, leading to abrupt expansions and contractions of inertial bubbles that cannot be accurately represented linearly.

In 2012, Louisnard proposed a simple yet powerful nonlinear Helmholtz equation for the sound transmission through cavitating bubbly liquids [19]. This equation precisely correlates the imaginary part of the square of the wave number with the energy dissipated by cavitation bubbles, as a function of the acoustic pressure amplitude. However, it did not resolve the real part of the wave number, approximating it to the linear value proposed by Commander and Prosperetti [17], which is independent of the pressure amplitude. In 2018, Trujillo completed Louisnard’s model by correlating the real part of the square of the wave number with the acoustic pressure amplitude [20]. Subsequently, the model was used to predict the pressure field under a horn in an ultrasonic vessel, achieving a close approximation to experimental data [23]. New models, such as the one proposed by Sojahrood *et al.* in 2023 [21], have confirmed the pressure dependence of the real and imaginary parts of the wave number. They validated their model through better-controlled experiments. While modelling the sound transmission through cavitating liquids is not yet completely resolved, these models are closely predicting the interaction of cavitating bubbles with acoustic fields, and their strong attenuation.

The nonlinear Helmholtz model developed by Louisnard and Trujillo reads as:

(4) $$\nabla^{2}P+k_{m}^{2}\;P=0$$

where P is a complex acoustic pressure amplitude and $k_{m}^{2}$ is the complex square of the wave number, defined as

(5) $$k_{m}^{2}\;=R\left(k_{m}^{2}\;\right)+iJ\left(k_{m}^{2}\;\right)$$

Where $R\left(k_{m}^{2}\;\right)$ and $J\left(k_{m}^{2}\;\right)$ are the real an imaginary part of $k_{m}^{2}$:

(6) $$R\left(k_{m}^{2}\;\right)\;\;=\;\frac{\omega^{2}}{c_{l}^{2}}-\frac{A}{\left|P\right|}$$

(7) $$J\left(k_{m}^{2}\;\right)=-\frac{B}{\left|P\right|}$$

where $c_{l}$ is speed of sound in the liquid, ω=2πf is the angular frequency, f is frequency, and $\left|P\right|$ is the modulus of the complex pressure P. A And B were defined as

(8) $$A=-2\rho_{l}\omega^{2}\langle \frac{\partial^{2}\beta }{{\partial \tau }^{2}}cos\left(\tau +\pi /2\right)\rangle =-2\rho_{l}\omega^{2}\langle \frac{\partial \beta }{\partial \tau }sin\left(\tau +\pi /2\right)\rangle$$

(9) $$B=2\rho_{l}\omega^{2}\langle \frac{\partial^{2}\beta }{{\partial \tau }^{2}}sin\left(\tau +\pi /2\right)\rangle =\frac{{2\rho }_{l}\omega }{\left|P\right|}\langle \Pi_{\mathrm{dis}}\rangle$$

where $\rho_{l}$ is the liquid density, τ=tω is the dimensionless time expressed in radians, t is time, β is the volumetric fraction of bubbles (also called void fraction), $\langle \Pi_{\mathrm{dis}}\rangle$ is the time averaged energy dissipation density. The brackets 〈〉 represent a time average over a period. The speed of sound through the bubbly mixture $c_{m}$ was defined as

(10) $$c_{m}=\frac{\omega }{k_{m}}$$

And the complex wave number as

(11) $$k_{m}=k_{r}-i\alpha$$

where $k_{r}$ is defined as the real par of $k_{m}$ and the sound attenuation α, in units of m^−1^. The terms A And B were estimated with a bubble dynamics model. Here, the Keller and Miksis equation (KME) was used. Argon bubbles with equilibrium bubble radius of $R_{o}$ = 3.00 × 10^−6^ were assumed to compare with Trujillo (2020) [23]. It was assumed that bubbles were homogenously distributed within the vessel having a constant bubble density $N_{\mathrm{tot}}=3\times 10^{9}$ bubble $m^{-3}$. Further details to solve the Helmholtz equation above are provided by these studies [20], [23].

#### 2.3.3 Acoustic streaming modelling

Acoustic streaming is the steady flow induced by the absorption of acoustic energy during the passage of acoustic waves [24]. During acoustic cavitation, the energy of the sound wave is dissipated by the inertial bubbles due to three mechanisms: viscous, thermal and acoustic radiation [25]. The acoustic force per unit of volume can be obtained from the acoustic velocity distribution as:(12)
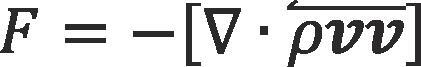
where v is the vector velocity of the acoustic field, obtained from the solution of the nonlinear Helmholtz equation (4) as $v=-\left(i/\omega \rho_{l}\right)\nabla p$, vv is the dyadic tensor product of v and v,  (a tensor) is the time average over a period of vv times density (ρ),  is the gradient tensor of , $p=p_{0}\left[1-P^{\prime }\sin\left(\omega \tau \right)\right]$ is the acoustic pressure field, $P\prime =\left|P\right|/p_{0}$ is the dimensionless acoustic pressure amplitude, $\left|P\right|$ is the amplitude of the acoustic wave, which is calculated as the norm of the complex pressure amplitude P, and $p_{0}$ is the static or time-averaged pressure.

The force described by Eq. (12) can be integrated into the Navier-Stokes equation as a volumetric force, inducing fluid flow (acoustic streaming). Louisnard modeled acoustic streaming in a cavitating system by considering the energy dissipation of inertial bubbles [26]. In this study, the forces causing acoustic streaming were estimated from the attenuated pressure field resulting from the energy dissipated by the bubbles.

### 2.4 Loading percentage

We defined Basket Loading Percentage (BLP) as the mass of coffee in grams divided by the loading capacity of the filtering basket in grams. The latter is provided by the manufacturers of espresso coffee baskets and is generally understood as the grams of ground coffee that will fill the basket to the top without pushing down or tamping the coffee. The effects of the BLP on physical properties, such as pH, total titratable acidity (TA), color, extraction yields, as well as on the content of caffeine, fatty acids, and volatiles, were determined. The BLP varied from 33 % to 100 %. Studies were conducted with a 2-cup single-wall 58 mm Breville filter basket, with a loading capacity of 18 g of coffee.

### 2.5 Extraction yields (EY) and total titratable acidity

The EY of the brewed coffee was determined following the method outlined by Gloess *et al.* [27]; however, no filtration with a paper filter, as recommended by The Coffee Brewing Handbook – SCAA [28], was conducted in this study. TA was measured by titrating each coffee sample with a 0.025 mol/L NaOH aqueous solution until it reached both a pH of 6 and a pH of 8, using 10 mL of the sample [5].

### 2.6 Colorimetric analysis

The colorimetric analysis of sonicated and unsonicated coffee brews was carried out with a ColorQuest XE tristimulus spectrometer (Hunter Associates Laboratory Inc., Reston, VA) to measure the tristimulus L*, a*, and b* values, where L* represents the degree of lightness, a*, the degree of redness (positive value) or greenness (negative value), and b* the degree of yellowness (positive) or blueness (negative) [29]. The parameters were adjusted to match daylight illumination (D65) and the standard observer with a 10-degree field of view. The samples were pipetted into a 12.5 × 12.5 mm standard cuvette, which was then clamped into a 0.375-inch area in the spectrometer for measurements. Each sample was performed in triplicate and the averages and standard deviations were used to calculate the chroma and color intensity [30].

### 2.7 Caffeine measurement

Caffeine analysis was carried out by Nuclear Magnetic Resonance (qNMR) spectroscopy. We followed the method outlined by Bosco, Toffanin, Palo, Zatti and Segre [31], and Zamanipoor [12], with slight modifications. 1 mL of the extract was mixed with chloroform-D (1 mL) in a 15 mL falcon tube. The sample was vortexed at maximum speed for 5 min and centrifuged at 5000 rpm in a Thermoline Scientific centrifuge for 20 min. The chloroform-D layer at the bottom of the tube was collected using a glass syringe. The remaining sample underwent a second extraction process: it was mixed with chloroform-D (1 mL), vortexed, centrifuged, and the chloroform-D layer was then collected. To concentrate the sample prior to the NMR analysis, samples were dried by a nitrogen blow and then dissolved with chloroform-D (700 μL). Next, the chloroform layer (700 μL) was transferred into a 5 mm NMR tube and subjected to centrifugation in an NMR centrifuge at maximum speed for 5 min. NMR analysis of the extracted samples was performed using a Bruker Avance III HD 600 NMR spectrometer with a ^1^H frequency of 600.13 MHz. The equipment was equipped with a 5 mm TCI cryoprobe and a SampleJet auto-sampler. The ^1^H spectral parameters were set to a sweep width of 9009 Hz, an acquisition time of 3.64 s, and a recycle delay of 25 s. CDCl_3_ was used as the solvent for all samples, and the residual ^1^H solvent signal at δ_H_ 7.26 ppm was used as the reference. Data acquisition and processing were conducted using Bruker Topspin 4.2.0 software. The quantification of caffeine content (δ_H_ 3.42, 3.60, or 4.01 ppm) [12], [31] was carried out utilizing the ERETIC2 system within the Topspin software, employing an external reference of sodium acetate trihydrate dissolved in deuterium oxide (D_2_O) at a concentration of 50 mmol/L.

### 2.8 Fatty acids measurement

In every coffee species, fatty acids (FAs) appear as a combination of free fatty acids and fatty-acid-triglycerides. The primary fatty acids include palmitic acid (C_16_), and linoleic acid (C_18:2_), with significant or detectable amounts of stearic acid (C_18_), oleic acid (C_18:1_), arachidonic acid (C_20_), and docosanoic acid (C_22_) [4]. In this study, measuring total fatty acids was preferred instead of reporting the content of triglycerides, which is more complex to accurately determine. To confirm the fatty acids, present in our coffee samples, high-resolution mass spectra (HRMS) with electron spray ionization in both positive and negative ion modes were used at the Bioanalytical Mass Spectrometry Facility at UNSW. By checking the results obtained from HRMS, it was confirmed that four fatty acids (C_16_, C_18:2_, C_18:1_, and C_18_) were detectable and quantifiable in our coffee samples.

Fatty acids were converted into fatty acid methyl esters (FAMEs) [32], followed by gas chromatography–mass spectrometry (GC–MS) quantification. A calibration curve was constructed by converting the four FA standards to FAMEs. That calibration curve was used to quantify the FAMEs in the coffee samples. To construct the calibration curve, 50 µL of each of the FAs (C_16_, C_18:2_, C_18:1_, and C_18_) at concentrations of 0, 0.3, 1, 3, and 5 mg/mL were mixed with a 2 mg/mL solution of heptadecanoic acid (C_17:0_) (50 µL) in 13 mm/7 mL glass culture tubes, respectively. An odd-chain fatty acid, heptadecanoic acid, was selected as the internal standard due to its rarity and infrequent occurrence in most biological systems [33]. Subsequently, deionized water (900 µL) was added to each of the four fatty acid solutions to mimic the coffee aqueous solution environment. Chloroform (1 mL) was added, and the mixture was rigorously mixed using a mixer (RM-2L, ELMI) at a u2 mode with 90 RPM for 15 min. The solution was separated into two layers due to immiscibility, with the chloroform layer settling at the bottom. This chloroform layer was then collected with glass syringes and transferred to clean culture tubes. To remove the extraction solvent, the culture tubes containing the samples were dried under a stream of nitrogen gas, following the same procedure as NMR sample preparation. Next, a 0.5 M NaOH (300 µL) solution prepared in dry methanol was added to each tube. The tubes were securely sealed with teflon-lined caps and heated to 70 °C for 12 min to facilitate the hydrolysis of triglycerides. After cooling, a solution of 14 % boron trifluoride (400 µL) in dry methanol was added to the tubes. The tubes were re-capped and heated to 100 °C for 7 min. Following another round of cooling, heptane (2 mL) was added to each tube. Intense agitation was applied for 10 min to extract the FAMEs. Next, water (2 mL) was added, and further agitation helped remove the remaining reagents from the heptane layer into the underlying aqueous layer. The samples were then subjected to centrifugation (1,100g, 15 min), and the upper heptane layer was carefully poured off into clean 7 mL tubes.

A volume of 0.5 μL of the FAMEs mixture was injected into the heated inlet (240 °C) of a Thermo TSQ Quantum XLS gas chromatograph connected to a mass spectrometer (Thermo Scientific, Andover, MA). The inlet was equipped with a silicone/PTFE septum (Thermo AsepT) and an inlet liner specifically designed for split-mode operation. Separation of the FAMEs was achieved using an HP–88 column (60 m × 0.25 mm, 0.2 μm film thickness, 112–8867, Agilent). Helium gas was the carrier gas, flowing at a constant rate of 1.3 mL/min, with a split flow of 80 mL/min during injection. The GC oven was initially held at a temperature of 40 °C for 1.5 min after injection. Then, the temperature was gradually increased to 100 °C, 150 °C, and finally 200 °C at rates of 15, 10, and 3 °C/min, respectively. Subsequently, the temperature underwent a further increase to 235 °C at a rate of 10 °C/min, where it was maintained for 7 min. The GC transfer line was maintained at 245 °C, while the ion source of the mass spectrometer was set to 200 °C. The mass spectrometer scanned a range of *m*/*z* 35–500 at a rate of 2.5 scans per second. The compounds were identified by comparing their retention times with those obtained from analysing the standard mixture of C_8_-C_24_ FAMEs (CRM18918, Supelco) and/or by comparing their averaged, background-subtracted mass spectra with spectra from the combined Wiley 9/NIST 11 mass spectral library.

### 2.9 Headspace volatiles measurement

The volatile compounds on both sonicated and unsonicated coffee samples were identified and analyzed with headspace solid-phase microextraction (HS–SPME) gas chromatography coupled with mass spectrometry (GC–MS). The HS–SPME–GC–MS analysis was slightly modified from the method described by Córdoba *et al.* [34]. A Thermo Trace DSQII gas chromatographer with a Thermo Triplus SPME (Thermofisher Scientific, MA, USA) and a triple quadrupole mass spectrometer were used. The fiber (65 µm, 5191–5873, Agilent) was made of divinylbenzene/polydimethylsiloxane (DVB/PDMS). 1 mL of coffee sample was transferred into a 10 mL sealed glass GC vial for both sonicated and control samples. These vials were then equilibrated with the fiber for 1 h at room temperature of ∼ 22 °C. Subsequently, the samples were injected into the GC–MS system with a desorption time of 5 min at 250 °C. During the analysis, a DB-FFAP column (60 m × 0.25 mm, 0.5 μm film thickness, 122–3263, Agilent) was used. The oven temperature was initially set at 45 °C and gradually increased at a rate of 5 °C/min until reaching 250 °C. The injector temperature was programmed at 250 °C in splitless mode, using helium as the carrier gas. To condition the fiber for the next sample run, a split flow of 100 ml/min was used for 2 min. For the MS, the ion source was set to 70 eV and 230 °C. The mass-to-charge ratio (*m*/*z*) range for MS scanning was set from 30 to 350 in positive ion mode, with a scanning rate of 6 scans per second.

### 2.10 Structural analysis

The surface morphology of both sonicated and unsonicated spent coffee samples (i.e., residual coffee grounds after brewing) was examined via scanning electron microscopy (SEM, JEOL JSM-IT 500 HR). Spent coffee samples were collected from the coffee basket after brewing. Samples were stored at a temperature of –20 °C to preserve their morphology and undergo a freeze–drying process for approximately 24 h. Then, 1 mg of sample was affixed to a silicon substrate using a conductive tape. To ensure optimal SEM imaging quality for materials with lower conductivity, the dried coffee samples were coated with a ∼ 5 mm layer of platinum through a sputtering process, enhancing the observation capability.

### 2.11 Sensory analysis

Sensory experiments were conducted on three samples: the first one was sonicated at room temperature for 1 min, the second was sonicated for 3 min, and the third was unsonicated and brewed at 4 °C for 24 h (traditional cold brew). All samples were prepared with a brew ratio equal to 10, and they were collected and stored at −19 °C for approximately 2 weeks before sensory evaluation.

The sonicated samples were prepared with ambient temperature water by applying 100 W of ultrasound power at a frequency of 38.8 KHz. Five extractions were performed during the brewing time (1 or 3 min), with each extraction lasting around 4 s, and taking place every 12 and 36 s, for the brewing times of 1 and 3 min, respectively. For the cold brew, coffee grounds were placed in a jar and then placed in a 4 °C refrigerator for 24 h. 6 L of each sample were produced for the sensory analysis.

Frozen samples were sent to the Queensland Alliance for Agriculture and Food Innovation (QAAFI) at the University of Queensland (UQ) for sensory analysis. At arrival, samples were transferred to a refrigerator (2 °C) approximately 48 h before each sensory session and allowed to reach room temperature (23 °C) approximately 3 h before the tasting began. Each sample was vigorously shaken and poured into 4 oz single-wall hot cups 1.5 h before serving. A watch glass lid was placed on top of each cup to contain the volatile compounds. Three samples were served simultaneously at room temperature.

This research adheres to the National Statement on Ethical Conduct in Human Research and was approved by the Sub-Committee for Human Research Ethics of the University of Queensland under project number 2023/HE001242. Informed consent was obtained from panellists prior to participation in the sensory study, and participants were paid for their time. A total of eleven trained sensory panellists participated in the study, comprising 8 females and 3 males, with ages ranging from 22 to 52 years. These panellists were selected from a group of experienced assessors who had previously been screened for their sensory acuity. The study followed a conventional quantitative sensory descriptive analysis method to characterize the sensory properties. It consisted of eight sessions, including four training sessions, one practice formal assessment, and three formal assessments. The sessions extended for up to 2 h over a period of 2 weeks. The training encompassed tasks such as acquainting the panellists with the samples, developing an assessment methodology, creating a vocabulary to describe the sensory properties of the samples, defining sensory attributes, establishing corresponding sensory reference standards, and devising scales and anchors for rating these attributes. The samples were selected randomly from each treatment group daily, with an approximately equal distribution of treatments each day. During the formal evaluation sessions, panellists were instructed to review the attribute definitions and reassess the sensory reference standards before evaluating the samples.

The sensory properties rated included 8 aroma (aroma intensity, aromatic, fruity, cream, dark caramel, dark chocolate, nutty and ashy), 1 texture (fullness), 6 flavour (flavour intensity, sourness, saltiness, sweetness, bitterness and ashy) and 4 aftertaste attributes (sourness, bitterness, ashy, and astringency). Attributes ‘other aroma’, ‘other texture’, ‘other flavour’ and ‘other aftertaste’ attributes were also included for panellists to rate and describe if any other sensory property was perceived during tasting. The sensory attributes, together with their definitions and composition of the sensory reference standards are detailed in Table S2 (Supporting Information). All attributes were rated on unstructured line scales ranging from 0 to 100, with anchors from “none” to “high”. Within each 2-hour session, a maximum of 9 samples were presented, with mandatory 2 min breaks between samples. Three samples were presented at a time, followed by a 5–10 min break after each set of 3 samples. Data were collected electronically using the Redjade software (Redjade Software Solutions, LLC, Tragon Corporation, California, USA, 2019).

All training sessions were conducted in a meeting room equipped with an electronic whiteboard. Practice and formal evaluation sessions were held in the purpose-built sensory evaluation laboratory of QAAFI located at the Elkhorn building (UQ Long Pocket Campus, Indooroopilly). The sensory evaluation area consists of 14 isolated sensory booths, tablets, temperature control (22 °C), and under day-light equivalent lighting.

Data was exported from RedJade into Microsoft Excel for analysis by XLSTAT (Version 5). For all sensory attribute scores, minimum, maximum, mean, standard deviation, and coefficient of variation were calculated. Sample data were also plotted on a cobweb plot to aid in the interpretation of differences observed.

## 3 Results and discussions

### 3.1 Simulation results

**Fig. 3:**
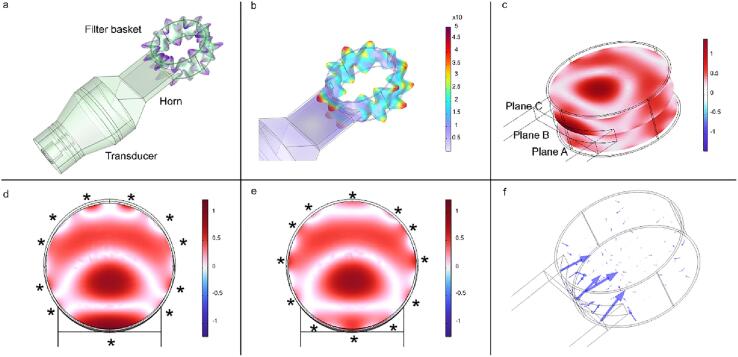
Simulation results. At a frequency of 38,280 Hz, all figures depict results, with a representing an eigenfrequency simulation, while b to f showcase frequency domain simulations. a) An eigenfrequency simulation showing resonance at 38,280 Hz. b) Total displacement with deformation amplifying details around the coffee filter basket (the maximum deformation depicted in the simulations is of the order of 1 Mpa. Given that the yield strength of 316 stainless steel, the material of the coffee basket, is 205 MPa, the coffee basket is not expected to experience permanent deformation). c) Three horizontal planes A, B, and C in the coffee basket display the dimensionless acoustic pressure P′. d) Dimensionless acoustic pressure P′ in plane B (centre plane). e) dimensionless acoustic pressure P′ in plane C (top plane). f) velocity vectors of the acoustic force that causes acoustic streaming.

The model couples vibration on the metallic parts, piezoelectricity, and acoustic cavitation inside the basket as explained in the Methods section. The force field responsible for acoustic streaming was calculated. The results are illustrated in Fig. 3, where Fig. 3a displays an eigenfrequency simulation of the vibration and deformation of the entire unit, including the transducer, horn, and coffee filter basket. The complete unit resonates at 38,280 Hz, generating resonant waves around the walls of the coffee basket. The modelled resonance frequency is slightly below the experimental frequency of 38,800 Hz, which was determined with a Network Analyzer. Fig. 3b amplifies the details of the displacement of the acoustic waves around the filter basket. Fig. 3c shows the dimensionless acoustic pressure P′ on three planes in the coffee baskets as Planes A, B, and C. Fig. 3d shows the dimensionless acoustic pressure on the centre plain. The asterisks demark the points of high acoustic amplitude from which sound can be injected into the basket. The regions where acoustic cavitation may occur are depicted with intense red colour. The bottom of the figure corresponds to the contact of the basket with the horn, where maximum pressure amplitude is achieved. Fig. 3e shows P′ on the top plane. In this and other planes where the horn does not contact the coffee basket directly, additional points of high-pressure amplitude are formed around the edges, from which sound can be injected. Those asterisks correspond to the peaks of maximum displacement shown in Fig. 3a,b. Fig. 3c,d,e show an inner section of high acoustic intensity, where acoustic cavitation may also occur. The last Fig. 3f shows the vector field of the force responsible for acoustic streaming. It shows that greater force is formed at the point of contact with the horn. Video S1 shows the acoustic streaming generated in a basket filled with coffee grounds and water, in a disengaged position from the coffee machine.

**Fig. 4:**
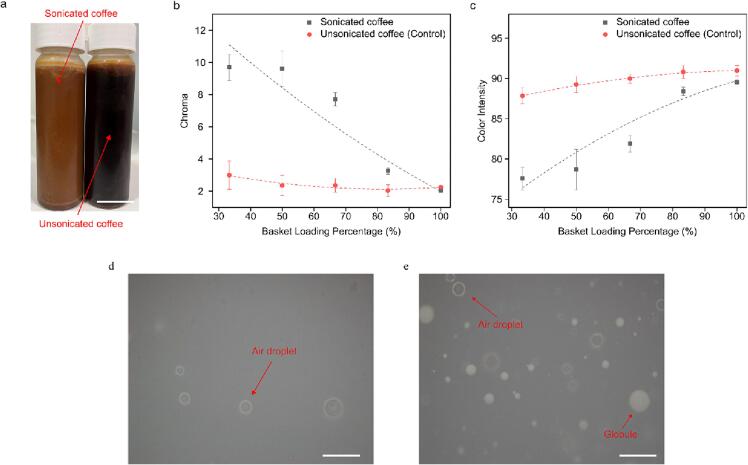
Color intensity and chroma difference *vs* basket loading percentage (BLP) for sonicated and unsonicated coffee brews. a) Appearance (scale bar: 2.5 cm). b) Chroma. c) Color intensity. For both graphs, values are means ± s.d. (n = 3 independent tests). Images of coffee samples taken by a light microscope (scale bar: 20 µm). d) unsonicated coffee. e) sonicated coffee.

**Fig. 5:**
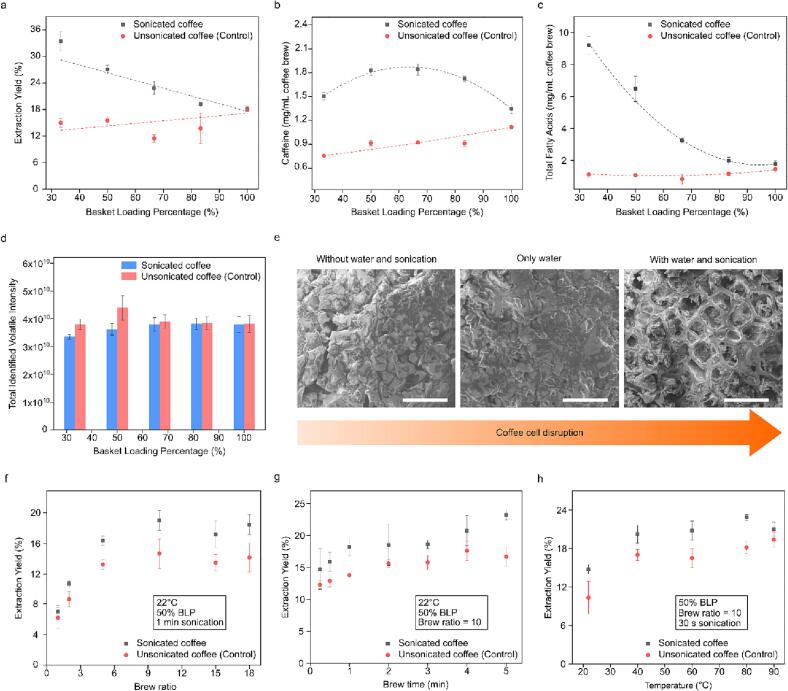
The effect of ultrasonication on coffee by changing different processing parameters. a-d) Caffeine concentration, total fatty acid concentration, and total volatile intensity *vs* basket loading percentage. e) SEM images of sonicated and control coffee grounds (scale bar: 50 µm). f-h) Extraction yield *vs* brew ratio, brewing time, and temperature. For all graphs, values are means ± s.d. (n = 3 independent tests).

The right-hand side of Fig. 2 illustrates a hypothetical mechanism of the brewing process. Ultrasound, injected throughout the walls of the coffee basket, induces acoustic cavitation, causing large coffee particles to be pitted and the subsequent release of small coffee particles into the water. The pitting is observed in the scanning electron microscopy images in Fig. 5e. Meanwhile, oils trapped in the coffee are released from the ground coffee and emulsified, as evidenced by the light microscopy images in Fig. 4e. The small particles pitted from the large coffee grounds accelerate the dissolution of water-soluble material, resulting in a brew with increased dissolved solids, but also containing undissolved small coffee particles and emulsified oils.

Our model grasps the effect of acoustic cavitation inside the filter basket and demonstrates that, firstly, the sound transmission unit, composed of the transducer, horn and coffee basket, achieves resonance, as shown by the eigenfrequency simulation. Secondly, the resonant waves circulating around the filter basket enable multiple sound injections into the basket. Thirdly, there are multiple regions, close to the walls of the coffee basket and an inner region in the basket, where acoustic cavitation may occur. Lastly, the attenuation of the acoustic field generates volumetric forces that induce acoustic streaming, increasing mixing, and consequently, enhancing mass transfer during brewing. These factors collectively transform the coffee basket into a potent ultrasonic reactor, significantly accelerating the brewing process, and proving that our modelling approach can be used for the design of sonorectors, taking advantage of resonance, acoustic cavitation, and acoustic streaming, for a fast and effective brewing of coffee, and other extractions, as demonstrated in subsequent parts of this paper.

### 3.2 Effect of loading percentage

Preliminary experiments revealed a strong correlation between the fullness of the brewing basket with coffee grounds and both the quantity of extracted components and physical properties of the brew. The less the basket is filled with coffee, the more fluid the water-coffee mixture enhances the extractive effects of acoustic cavitation. The basket loading percentage (BLP), defined in the methods section, represents how full the basket is with coffee. Hence, we conducted experiments to investigate the effects of the BLP on the physical properties of coffee brews, including pH, total titratable acidity (TA), color, extraction yields, as well as the content of caffeine, fatty acids, and volatiles.

Table S1 presents the effect of the BLP on pH and total TA at pH levels of 6.0 and 8.0. The pH for all samples was ∼ 5.1, independent of the BLP. No significant differences in pH were observed between the sonicated and control samples, while significant differences in TA at pH levels of 6.0 and 8.0 were noted between sonicated and unsonicated samples.

There were differences in the appearance of coffee samples (Fig. 4a). Except for a BLP of 100 %, the chroma (Fig. 4b) was significantly higher for sonicated samples than for the control. Meanwhile, the color intensity (Fig. 4c) was significantly lower for sonicated samples. The chroma increased while the color intensity decreased with the decrease of the BLP. Sonicated coffee samples displayed brown caramel-like hues, while unsonicated samples looked darker in color. This was attributed to the emulsification of oils due to sonication. The color differences between sonicated and control samples grew rapidly with the decrease of the BLP. The maximum differences were observed at the lowest BLP of 33 %, with chroma exhibiting its highest value (9.70 ± 0.82) compared to the control (2.04 ± 0.10), and color intensity reaching its minimum (77.6 ± 1.4) compared to the control (87.8 ± 1.0).

The observed color changes can be attributed to the ultrasonic emulsification of coffee oil, which increases as the basket loading percentage decreases. Zanin *et al.* [35] reported that ultrasound enhances the emulsification and stabilization of coffee oils. Light microscopy examination of both control (Fig. 4d) and sonicated (Fig. 4e) coffee samples revealed the presence of emulsified oil globules after sonication.

The effects of emulsification on color perception have been studied [36], [37]. It is suggested that the appearance of color in an emulsion is influenced by the relative proportions of light reflected and transmitted at different wavelengths [38]. Chroma and light intensity exhibit an inverse relationship. Below a certain relative refractive index, chroma increases while light intensity decreases. This effect is intensified with an increase in droplet concentrations and radii. Droplet concentration is proportional to the content of fatty acids. These trends align with the observations in Fig. 4b and 4c.

The extraction yield (EY) of sonicated samples also exhibited significant differences compared to the control samples (Fig. 5a). The EY of sonicated samples increased from 18.04 % to 33.44 % when decreasing the BLP from 100 % to 33 %, respectively. The highest EY of coffee solids, 33.44 % ± 2.09 % corresponded to the lowest BLP of 33 %. The EY of control samples slightly decreased from 18.15 to 15.05 when decreasing the BLP from 100 % to 33 %, respectively.

The caffeine concentration was higher in sonicated than in control samples, with concentrations ranging between 1.34 and 1.84 mg/mL, while the concentration of control samples ranged from 0.74 to 1.1 mg/m (Fig. 5b). This shows that ultrasound enhanced the extraction of caffeine in the brew. Caffeine concentration in sonicated samples increased from 1.34 mg/mL to a maximum of 1.84 mg/mL when decreasing the BLP from 100 % to 67 %, respectively. Subsequently, the caffeine concentration decreased from its maximum to 1.50 mg/mL while further decreasing the BLP from 67 % to 33 %.

The concentration of fatty acids represents the content of coffee oils in the brew. Four fatty acids: palmitic acid C_16_, linoleic acid C_18:2_, oleic acid C_18:1_, and stearic acid C_18_, were observed and quantified in the coffee brews. These specific fatty acids are commonly employed for coffee quality comparison, given their significance as key components in coffee [39]. Fig. 5c displays the total concentration of fatty acids by adding the concentration of the four fatty acids above. The concentration of fatty acids in sonicated samples was higher than in the control, and they increased when the BLP was decreased from 100 % to 33 %. Meanwhile, the fatty acid concentration of the control (unsonicated) remained almost constant, hovering around an average value of 1.16 mg/mL, when the BLP was decreased from 100 % to 33 %. This aligns with the trend exhibited by the extraction yield, indicating that ultrasound is more effective for extraction at lower basket loading percentages. This is because the effects of acoustic cavitation increase with the percentage of water in the basket.

The volatile profile observed in both coffee brews, sonicated and unsonicated, comprised 32 compounds, including furans and pyrazines, consistent with the analysis performed by Cordoba *et al.* [34]. Additionally, minor classes of volatile compounds, such as ketones, pyrroles, aldehydes, furanones, and phenols, were also detected (Table 1). The predominant compounds were furans. Among these, 5-Methylfuran-2-carbaldehyde (5-methylfurfural), Furan-2-yl-methyl-acetate (2-furanmethanol acetate), (Furan-2-yl)methanol (2-furanmethanol), and Furan-2-carbaldehyde (furfural) were consistently present at levels exceeding 10 % in every sonicated brew with different basket loading percentages. Notably, when considering the odor activity value, 2-furanmethanol acetate, known for its fruity, ester-like, roast, and sweet characteristics, along with 5-methylfurfural and 2-furanmethanol, were the most prominent among all volatile compounds. These compounds have been identified as key furans contributing significantly to the aroma profile, particularly the sweet, fruity, bread-like, roast, and caramel notes in hot coffee beverages, as discussed by Amanpour & Selli [40].

**Table 1:** The relative percentage of 32 identified volatile compounds in sonicated coffee under the different basket loading capacities.

Basket loading capacity	33.3 %	50 %	66.7 %	83.3 %	100 %	Aroma descriptor
Compound						
Butanal	0.33 ± 0.01	0.35 ± 0.01	0.42 ± 0.05	0.45 ± 0.02	0.46 ± 0.08	Chocolate, cocoa [44]
3-Methyl-furan	0.87 ± 0.15	0.91 ± 0.18	0.81 ± 0.17	0.77 ± 0.08	0.88 ± 0.16	−
Butan-2-one	0.15 ± 0.01	0.16 ± 0.01	0.17 ± 0.01	0.19 ± 0.01	0.18 ± 0.02	Sweet reminiscent of acetone [44]
2-Methyl-butanal	2.24 ± 0.23	2.45 ± 0.11	2.93 ± 0.29	3.14 ± 0.10	3.10 ± 0.20	Buttery [45]
3-Methyl-butanal	1.11 ± 0.08	1.23 ± 0.06	1.41 ± 0.14	1.54 ± 0.03	1.45 ± 0.06	Buttery [45], Fruity, malty [46]
Pentane-2,3-dione	0.95 ± 0.03	0.99 ± 0.03	0.98 ± 0.10	1.00 ± 0.06	0.92 ± 0.08	Buttery, creamy, sweet [44], [46]
2-Methylhexan-3-one (Methyldisulfanyl)methane	0.29 ± 0.03	0.27 ± 0.04	0.23 ± 0.03	0.21 ± 0.02	0.22 ± 0.03	Moldy [44]
Hexanal	0.26 ± 0.08	0.15 ± 0.02	0.12 ± 0.01	0.12 ± 0.01	0.11 ± 0.01	Green, grassy, fruity [44], [45]
2-Methyl-3-hexanone	0.29 ± 0.02	0.30 ± 0.02	0.30 ± 0.03	0.29 ± 0.02	0.26 ± 0.02	Fruity [44]
Hexane-3,4-dione	0.36 ± 0.02	0.35 ± 0.02	0.36 ± 0.02	0.34 ± 0.04	0.30 ± 0.03	Buttery, cooked, caramel [47]
1-Methyl-1H-pyrrole	0.46 ± 0.06	0.44 ± 0.04	0.42 ± 0.05	0.37 ± 0.03	0.33 ± 0.06	Woody [44]
Pyridine	3.36 ± 0.19	3.37 ± 0.35	3.16 ± 0.15	2.90 ± 0.13	2.72 ± 0.3	Bitter plant like [48]
2-Methoxymethyl-furan	0.85 ± 0.06	0.81 ± 0.05	0.90 ± 0.06	0.94 ± 0.05	0.90 ± 0.08	Mustard [44]
Dihydro-2-methyl-3(2H)-furanone	1.93 ± 0.28	1.83 ± 0.32	1.82 ± 0.26	1.74 ± 0.32	1.73 ± 0.27	Bread, nutty [44]
2,5-Dimethyl-pyrazine	1.85 ± 0.22	1.95 ± 0.22	2.15 ± 0.24	2.04 ± 0.22	2.20 ± 0.20	Hazelnut/roasted [45], nutty, roasted, grassy [46]
2,6-Dimethyl-pyrazine	1.76 ± 0.20	1.90 ± 0.22	2.09 ± 0.24	1.96 ± 0.20	2.09 ± 0.18	Chocolate, coca, roasted nuts [46]
Ethyl-pyrazine	1.77 ± 0.22	1.88 ± 0.22	2.03 ± 0.22	1.93 ± 0.17	2.01 ± 0.21	Peanuts/roasted [45], nutty, butter [46]
2,3-Dimethyl-pyrazine	0.42 ± 0.07	0.44 ± 0.06	0.49 ± 0.06	0.47 ± 0.05	0.49 ± 0.05	Hazelnut/roasted [45], nutty, roasted [46]
2-Ethyl-6-methylpyrazine	1.71 ± 0.18	1.91 ± 0.20	2.17 ± 0.27	2.08 ± 0.20	2.24 ± 0.23	Flowery, fruity, hazelnut-like [46]
2-Ethyl-5-methylpyrazine	1.15 ± 0.11	1.26 ± 0.13	1.46 ± 0.18	1.42 ± 0.16	1.52 ± 0.15	Coffee-like [46]
2-Ethyl-3-methylpyrazine	0.97 ± 0.11	1.08 ± 0.14	1.21 ± 0.16	1.15 ± 0.14	1.25 ± 0.13	Nutty, peanut [46]
1-(Acetyloxy)-2-propanone	1.41 ± 0.15	1.40 ± 0.15	1.48 ± 0.17	1.42 ± 0.19	1.45 ± 0.16	Fruity, buttery, dairy [46]
Furan-2-carbaldehyde (furfural)	13.94 ± 0.57	13.47 ± 0.70	13.39 ± 0.95	13.85 ± 0.99	13.46 ± 1.10	Cooked pea, chemical, smoky [49]
2-(Methylsulfanylmethyl) furan	1.18 ± 0.10	1.01 ± 0.13	0.85 ± 0.08	0.72 ± 0.04	0.62 ± 0.08	Cooked pea, chemical, smoky [49]
Furan-2-yl-methyl formate	3.36 ± 0.35	2.91 ± 0.35	2.87 ± 0.30	2.90 ± 0.12	2.44 ± 0.28	Ethereal [34]
1-(Furan-2-yl)-ethanone	4.38 ± 0.43	4.33 ± 0.37	4.63 ± 0.33	4.68 ± 0.44	4.68 ± 0.34	Grassy, plastic [49]
Furan-2-yl-methyl-acetate	10.51 ± 7.06	10.29 ± 6.91	5.90 ± 7.64	6.21 ± 8.02	6.22 ± 7.96	Ethereal-floral, herbal-spicy [46]
5-Methylfuran-2-carbaldehyde	16.08 ± 1.37	16.66 ± 1.41	18.42 ± 1.52	18.93 ± 1.84	19.23 ± 1.57	Spice, caramel [46]
1-Methylpyrrole-2-carbaldehyde	2.23 ± 0.43	2.37 ± 0.37	2.60 ± 0.36	2.72 ± 0.41	2.76 ± 0.40	Roasted, nutty [46]
(Furan-2-yl)methanol	18.39 ± 1.72	18.25 ± 1.31	18.89 ± 1.53	18.47 ± 1.98	18.69 ± 1.65	Burnt, smoky [46]
1H-pyrrole-2-carbaldehyde	1.14 ± 0.33	1.01 ± 0.14	1.10 ± 0.15	1.11 ± 0.12	1.10 ± 0.12	Vegetable, musty, beefy [46]
4-Ethenyl-2-methoxyphenol	0.85 ± 0.13	0.72 ± 0.11	0.64 ± 0.10	0.60 ± 0.08	0.61 ± 0.09	Phenolic/Clove [45]

The pronounced concentration of furans and pyrazines in the samples is probably responsible, at least partially, for the perception of sweetness, caramel notes, fruity hints, roasted qualities, and earthy undertones [41]. Volatile furans have been associated with the sweet, nutty, malty, roasted, and fruity flavour characteristics in coffee [42]. Furthermore, pyrazines are associated with the development of nutty, earthy, roasty, and green aromatic notes, and are one of the main groups affecting the complexity of coffee aroma and flavour [42]. The total intensity of volatile compounds in sonicated samples was not significantly different from that in the control for BLP values above 50 % (Fig. 5d). However, for BLPs lower than 50 %, the total intensity of volatile compounds slightly decreased in sonicated samples compared to the control. This may be due to the volatile compounds being extracted efficiently at lower loading percentages (as with EY, and fatty acids), with some compounds escaping from the coffee basket due to the agitation induced by acoustic vibration and streaming.

Scanning electron microscopy (SEM) showed the impact of ultrasonic cavitation on unsonicated and sonicated coffee grounds (Fig. 5e). In the sonicated coffee, distinct cavities are observed, indicating the erosion on the surface of coffee grounds caused by acoustic cavitation, which facilitates the extraction of compounds [43].

### 3.3 Extraction yields vs brew ratio, brewing time, and temperature

Coffee brewing is affected by various process parameters including brewing time, temperature, and brew ratio. Here, a 25 g ridgeless VST coffee basket (VST Precision Basket, USA) was used to study the effects of these parameters on the extraction yield. The brew ratio is the ratio of the produced brew to the dry coffee used. Fig. 5f shows the effect of brew ratios from 1 to 18 on the extraction yield. The brewing was performed at ambient temperature (22 °C), 50 % BLP, and sonicated for 1 min. Sonicated samples exhibited significantly higher extraction yield (EY) than the control in all cases. There was a significant increase in EY with the brew ratio in both sonicated and control samples. The highest EY was achieved at a brew ratio of 10, yielding 19.10 % ± 1.30 % and 14.73 % ± 1.94 % for sonicated and control samples, respectively. However, for brew ratios above 10, the EY showed no significant increase and reached a plateau.

Brewing time was varied from 15 s (0.25 min) to 5 min with ambient water temperature (22 °C), brew ratio of 10, and BLT of 50 %. Sonication increased the EY compared to the control. Increasing the brewing time increased the extraction yield. For instance, the sonicated EY increased from 14.74 % to 23.26 % when increasing the brewing time from 15 s to 5 min, respectively (Fig. 5g). In Fig. 5h, the temperature was varied from 22 °C to 90 °C with a constant brewing time of 30 s, brew ratio of 10, and basket loading percentage of 50 %. The EY significantly increased with the increase of the brewing temperature. For instance, in sonicated samples, the EY increased from 14.76 % to 22.86 %, while in control samples, it increased from 10.33 % to 19.38 % as the temperature increased from 22 °C to 90 °C, respectively. The application of ultrasound significantly increased the EY compared to the control at all temperatures, except at 90 °C, where there were no significant differences.

### 3.4 Sensory analysis

**Fig. 6:**
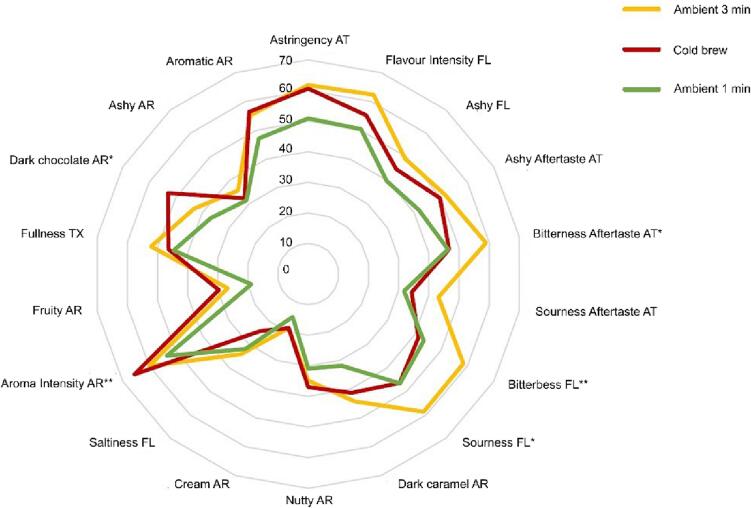
Cobweb plot for all attributes (n = 3 samples × 4 replicates × 11 panellists) including whether they were significant among the three samples (Significant difference between samples indicated by * (p < 0.05), ** (p < 0.01), *** (p < 0.001), scale 0–100).

Sensory analysis was conducted to compare the actual taste of the brews by panellists trained in coffee. The coffee sensory analysis evaluates coffee using the senses, considering properties such as appearance, aroma, texture, flavour, and aftertaste. Sensory experiments were conducted on three samples: (a) 1 min sonicated at ambient temperature, (b) 3 min sonicated at ambient temperature, and (c) unsonicated cold brew at 4 °C for 24 h. Details of the sensory methodology are given in the Methods Section. Fig. 6 displays the cobweb plot for the three samples. Samples sonicated for 1 and 3 min at ambient temperature exhibited similar characteristics to cold brew for 13 out of the 18 attributes shown in Fig. 5. There were significant differences in aroma intensity, dark chocolate aroma, sourness flavour, bitterness flavour, and bitterness aftertaste. Compared to the 24-hour cold brew, the sonicated 1-minute sample received similar ratings, especially for flavour (FL) and aftertaste attributes (AT), including bitterness AT, sourness AT, bitterness FL, sourness FL, fullness texture (TX), and ashy aroma (AR). However, it scored lower in aroma intensity and dark chocolate aroma. This suggests that the sonicated 1-minute sample is slightly under-extracted compared to the 24-hour cold brew. Meanwhile, the sonicated 3-minute sample provided a similar dark chocolate aroma and aroma intensity to the 24-hour cold brew. Indeed, Table S3 shows no significant differences in dark chocolate aroma and aroma intensity between the sonicated 3-minute and 24-hour cold brew samples. However, the sonicated 3-minute displayed more bitter and sour flavours and aftertaste. This suggests that the sonicated 3-minute sample is over-extracted compared to the 24-hour cold brew. Hence, it is expected that a sonication time between 1 and 3 min is ideal for creating a coffee comparable with 24-hour cold brew coffee, depending on the interest of customers.

## 4 Conclusion

In conclusion, we have successfully designed and implemented an ultrasonic brewing system that transformed the basket of an espresso machine into an effective sonoreactor. Coffee brews are easily produced, following similar steps to the production of espressos. Since the ultrasonic components do not come into direct contact with flowing water and ground coffee, the brewing process remains free from contamination, ensuring food safety.

This reactor remarkably reduced the brewing time from 24 h to about 3 min or less. This accomplishment stemmed from the development of a comprehensive model encompassing vibration, piezoelectricity, and acoustic pressure in the cavitating filter basket, including acoustic streaming. Our design induced acoustic streaming, enhancing extraction through effective mixing by increasing mass transfer during brewing. It transformed the filter basket into a powerful ultrasonic reactor capable of injecting sound waves at multiple points around the coffee basket, thereby generating multiple zones for acoustic cavitation within the reactor. The efficiency of the extraction increased by decreasing the basket loading percentage (BLP).

A comparison between sonicated and unsonicated samples revealed that the sonoreactor, operated at 100 W of ultrasonic power, doubled the extraction yield and caffeine concentration. Specifically, at a BLP = 33 %, the extraction yield increased from 15.05 % to 33.44 %, while the caffeine concentration rose from 0.91 to 1.84 mg/mL at BLP = 67 %. Meanwhile, at BLP = 33 %, the total fatty acids concentration increased from 1.16 mg/mL to 9.20 mg/mL, representing an eightfold increase.

The ultrasound-brewed coffee exhibits sensory attributes nearly identical to a standard 24-hour cold brew. This breakthrough opens the door for coffee shops and restaurants to produce on-demand brews comparable to 24-hour cold brew, eliminating the need for large semi-industrial brewing units and the associated requirement for extensive refrigeration space.

## CRediT authorship contribution statement

**Shih-Hao Chiu:** Writing – review & editing, Writing – original draft, Methodology, Investigation, Formal analysis, Data curation. **Nikunj Naliyadhara:** Writing – review & editing, Writing – original draft, Methodology, Investigation, Formal analysis, Data curation. **Martin P. Bucknall:** Methodology, Investigation. **Donald S. Thomas:** Methodology, Investigation. **Heather E. Smyth:** Writing – review & editing, Methodology, Investigation, Formal analysis, Data curation. **Jaqueline M. Nadolny:** Writing – review & editing, Methodology, Investigation, Formal analysis, Data curation. **Kourosh Kalantar-Zadeh:** Writing – review & editing, Supervision, Resources, Project administration, Funding acquisition, Conceptualization. **Francisco J. Trujillo:** Writing – review & editing, Supervision, Resources, Project administration, Methodology, Investigation, Funding acquisition, Formal analysis, Data curation, Conceptualization.

## Declaration of competing interest

The authors declare the following financial interests/personal relationships which may be considered as potential competing interests: Francisco J. Trujillo and Kourosh Kalantar-Zadeh has patent issued to NewSouth Innovations Pty Limited, Australian provisional patent application No. 2023903956 on December 6, 2023. If there are other authors, they declare that they have no known competing financial interests or personal relationships that could have appeared to influence the work reported in this paper.

## Data Availability

The data supporting the findings of this study can be obtained from the corresponding authors upon reasonable request. Source data are provided with this paper.
