# Supplementary material for: Coffee brewing sonoreactor for reducing the time of cold brew from several hours to minutes while maintaining sensory attributes
Source: Ultrason Sonochem. 2024 Apr 23;106:106885. doi: 10.1016/j.ultsonch.2024.106885 (PMC11061338; doi:10.1016/j.ultsonch.2024.106885)
Supplement: Supplementary Data 1 [file mmc1.docx]

**Supplementary materials**

**Coffee brewing sonoreactor for reducing the time of cold brew from several hours to minutes while maintaining sensory attributes**

*Shih-Hao Chiu ^a,e^, Nikunj Naliyadhara ^a^, Martin P. Bucknall ^b^, Donald S. Thomas ^c^, Heather E. Smyth ^d^, Jaqueline M. Nadolny ^d^, Kourosh Kalantar-Zadeh ^e *^ and Francisco J. Trujillo ^a *^*

^a^ School of Chemical Engineering, University of New South Wales, Sydney 2052, NSW, Australia

^b^ Bioanalytical Mass Spectrometry Facility, Mark Wainwright Analytical Centre, University of New South Wales, Sydney 2052, NSW, Australia

^c^ NMR Facility, Mark Wainwright Analytical Centre, University of New South Wales, Sydney 2052, NSW, Australia

^d^ Centre for Nutrition and Food Sciences, Queensland Alliance for Agriculture and Food Innovation, The University of Queensland, Brisbane 4072, Queensland, Australia

^e^ School of Chemical and Biomolecular Engineering, The University of Sydney, Sydney 2008, NSW, Australia

E-mail: [kourosh.kalantarzadeh@sydney.edu.au](mailto:kourosh.kalantarzadeh@sydney.edu.au); [francisco.trujillo@unsw.edu.au](mailto:francisco.trujillo@unsw.edu.au)

**Table S1**

pH and total titratable acidity measured at pH = 6 and pH = 8 versus basket loading percentage for both sonicated and unsonicated coffee (control).

| Basket loading percentage (%) | pH | | TA = 6 | | TA = 8 | |
| --- | --- | --- | --- | --- | --- | --- |
|  | Sonicated | Control | Sonicated | Control | Sonicated | Control |
| 33.3 | 5.07 ± 0.04 | 5.13 ± 0.02 | 3.97 ± 0.25 | 2.13 ± 0.12 | 7.50 ± 0.59 | 4.13 ± 0.05 |
| 50 | 5.09 ± 0.02 | 5.14 ± 0.03 | 3.50 ± 3.93 | 3.93 ± 0.12 | 7.10 ± 0.24 | 8.27 ± 0.29 |
| 66.7 | 5.14 ± 0.01 | 5.12 ± 0.02 | 3.40 ± 0.24 | 3.57 ± 0.12 | 7.03 ± 0.61 | 7.60 ± 0.22 |
| 83.3 | 5.13 ± 0.01 | 5.14 ± 0.01 | 4.07 ± 0.19 | 3.73 ± 0.12 | 8.30 ± 0.43 | 7.80 ± 0.22 |
| 100 | 5.17 ± 0.01 | 5.16 ± 0.01 | 4.47 ± 0.47 | 5.03 ± 0.05 | 9.63 ± 0.25 | 10.23 ± 0.09 |

**Table S2**

Sensory attributes and definitions used in the sensory descriptive study of cold brewed coffees.

| **Attribute** | **Definition** | **Reference Standard** |
| --- | --- | --- |
| **Aroma** | (none to high) |  |
| *aroma intensity* | The overall aroma intensity of the sample | na |
| *aromatic* | A fragrant, leathery or tobacco-like spice note, hint of orange peel. | Twinings English Breakfast Tea dried leaves (½ tsp), “leather” #35 wine Aromaster kit (2 drops of on cotton wool) |
| *fruity* | A dried fruity note, like raisins, dried figs. | Sunbeam Australian Raisins (two raisins), Angas Park soft & juicy Figs (¼ fig) |
| *cream* | A cream note, buttery. | Western Star Thickened Cream (½ tsp) |
| *dark caramel* | Aroma of dark caramel, dark brown sugar. | CSR Muscovado dk brown sugar (½ tsp) |
| *dark chocolate* | A chocolate note, cocoa-like. | Old Gold dark chocolate (old gold 70%) (1 tsp freshly grated) |
| *nutty* | Aroma of toasted nuts, hazelnut skins, roasted and woody. | Lucky natural hazelnuts (two nuts heated in the microwave immediately before serving). |
| *ashy* | A burnt ashy aroma, acrid. | Ash from a burnt banana leaf (½ tsp) |
| *‘other’ aroma* |  |  |
| **Texture** |  |  |
| *fullness* | Feeling of fullness or thickness of sample in the mouth | water (low), 1g/L CMC solution (high) |
| *‘other’ texture* |  |  |
| **Flavour** |  |  |
| *flavour intensity* | The overall flavour intensity of the sample |  |
| *sourness* | The sourness of the sample. |  |
| *saltiness* | The salty taste of the sample |  |
| *sweetness* | The sweetness of the sample. |  |
| *bitterness* | The bitterness of the sample. |  |
| *ashy* | A burnt ashy flavour, acrid. | as per aroma |
| *‘other’ flavour* |  |  |
| **Aftertaste** |  |  |
| *sourness aft* | Intensity of sourness lingering after swallowing sample. |  |
| *bitterness aft* | Intensity of bitterness lingering after swallowing sample. |  |
| *ashy aftertaste* | Intensity of ashy taste lingering after swallowing sample |  |
| *astringency* | The astringent, drying sensation in the mouth and cheek pouches. |  |
| *‘other’ aftertaste/feel* | Intensity of sourness lingering after swallowing sample. |  |

**Table S3.**

ANOVA summary and LS means of sensory data for ambient 3 min, ambient 1 min, and cold brew samples (different letters between rows indicate a significant difference between samples by Tukey LSD) (Significant difference between samples indicated by * (p<0.05), ** (p<0.01), *** (p<0.001), scale 0-100).

| Attribute | Amb 3 min | Cold Brew | Amb 1 min | Pr > F (Model) | Significant |
| --- | --- | --- | --- | --- | --- |
| ***aroma intensity AR*** | 60.1 ab | 65.5 a | 53.3 b | 0.009 | Yes |
| *aromatic AR* | 55.2 a | 56.4 a | 47.2 a | 0.204 | No |
| *fruity AR* | 26.7 a | 29.7 a | 18.9 a | 0.238 | No |
| *cream AR* | 18.8 a | 18.8 a | 14.9 a | 0.675 | No |
| *dark caramel AR* | 44.2 a | 41.3 a | 31.9 a | 0.124 | No |
| ***dark chocolate AR*** | 43.0 ab | 52.9 a | 36.7 b | 0.046 | Yes |
| *nutty AR* | 34.8 a | 36.9 a | 30.9 a | 0.600 | No |
| *ashy AR* | 35.7 a | 32.3 a | 31.4 a | 0.802 | No |
| *fullness TX* | 52.2 a | 46.3 a | 44.7 a | 0.458 | No |
| *flavour intensity FL* | 62.4 a | 55.2 ab | 50.4 b | 0.055 | No |
| ***sourness FL*** | 58.7 a | 46.5 a | 46.6 a | 0.029 | Yes |
| *saltiness FL* | 34.1 a | 24.3 a | 31.9 a | 0.253 | No |
| *sweetness FL* | 16.8 a | 15.7 a | 15.7 a | 0.965 | No |
| ***bitterness FL*** | 58.5 a | 41.5 b | 43.6 b | 0.002 | Yes |
| *ashy FL* | 49.2 a | 44.7 a | 39.8 a | 0.359 | No |
| *sourness aftertaste AT* | 43.2 a | 34.3 ab | 31.8 b | 0.057 | No |
| ***bitterness aftertaste AT*** | 59.0 a | 46.7 a | 46.4 a | 0.046 | Yes |
| *ashy aftertaste AT* | 51.7 a | 49.7 a | 41.8 a | 0.313 | No |
| *astringency AT* | 61.8 a | 60.6 a | 50.9 a | 0.151 | No |

**Video S1**. Acoustic streaming of a coffee water slurry in a filter basket.
